# Obesity, metabolic syndrome and cardiovascular prognosis: from the Partners coronary computed tomography angiography registry

**DOI:** 10.1186/s12933-017-0496-8

**Published:** 2017-01-25

**Authors:** Edward A. Hulten, Marcio Sommer Bittencourt, Ryan Preston, Avinainder Singh, Carla Romagnolli, Brian Ghoshhajra, Ravi Shah, Siddique Abbasi, Suhny Abbara, Khurram Nasir, Michael Blaha, Udo Hoffmann, Marcelo F. Di Carli, Ron Blankstein

**Affiliations:** 1 Non-Invasive Cardiovascular Imaging Program, Departments of Medicine and Radiology, Brigham and Women’s Hospital, Harvard Medical School, Boston, MA USA; 2 Cardiology Service, Division of Medicine, Walter Reed National Military Medical Center and Uniformed Services University of Health Sciences, Bethesda, MD USA; 3 Center for Clinical and Epidemiological Research, University Hospital, University of São Paulo, São Paulo, Brazil; 4 Division of Medicine, Walter Reed National Military Medical Center and Uniformed Services University of Health Sciences, Bethesda, MD USA; 5 Cardiac MR PET CT Program, Department of Radiology, Division of Cardiac Imaging, Massachusetts General Hospital, Harvard Medical School, Boston, MA USA; 6 Cardiology Division, Department of Medicine, Beth Israel Deaconess Medical Center and Harvard Medical School, Boston, MA USA; 7 Cardiothoracic Imaging Division, UT Southwestern Medical Center, 5323 Harry Hines Blvd., Dallas, TX 75390-9316 USA; 8 Center for Wellness and Prevention Research, Baptist Health South Florida, Miami, FL USA; 9 Johns Hopkins Ciccarone Center for the Prevention of Heart Disease, Baltimore, MD USA; 10 Cardiovascular Division, Brigham and Women’s Hospital, 75 Francis St, Boston, MA 02115 USA

**Keywords:** Obesity, Metabolic syndrome, Coronary computed tomography angiography, Coronary artery disease, Prognosis, Cohort

## Abstract

**Objective:**

To investigate the relationship among body mass index (BMI), cardiometabolic risk and coronary artery disease (CAD) among patients undergoing coronary computed tomography angiography (CTA).

**Methods:**

Retrospective cohort study of 1118 patients, who underwent coronary CTA at two centers from September 2004 to October 2011. Coronary CTA were categorized as normal, nonobstructive CAD (<50%), or obstructive CAD (≥50%) in addition to segment involvement (SIS) and stenosis scores. Extensive CAD was defined as SIS > 4. Association of BMI with cardiovascular prognosis was evaluated using multivariable fractional polynomial models.

**Results:**

Mean age of the cohort was 57 ± 13 years with median follow-up of 3.2 years. Increasing BMI was associated with MetS (OR 1.28 per 1 kg/m^2^, p < 0.001) and burden of CAD on a univariable basis, but not after multivariable adjustment. Prognosis demonstrated a J-shaped relationship with BMI. For BMI from 20–39.9 kg/m^2^, after adjustment for age, gender, and smoking, MetS (HR 2.23, p = 0.009) was more strongly associated with adverse events.

**Conclusions:**

Compared to normal BMI, there was an increased burden of CAD for BMI > 25 kg/m^2^. Within each BMI category, metabolically unhealthy patients had greater extent of CAD, as measured by CCTA, compared to metabolically healthy patients.

## Background

Atherosclerosis, obesity, metabolic syndrome (MetS), and diabetes mellitus are closely linked and constitute major health problems worldwide. Not only has the prevalence of obesity been increasing to epidemic proportions in the United States, but obesity is also causally related to most of the major cardiovascular (CV) risk factors, including high blood pressure, dyslipidemia and insulin resistance leading to metabolic syndrome and type-2 diabetes mellitus [1]. Due to accelerated systemic atherosclerosis and resultant high cardiovascular event rates among patients with type-2 diabetes, the World Health Organization has called for increased preventive efforts to stem the tide of increasing prevalence of type-2 diabetes, which occurs in association with obesity [2]. Beyond risk factor changes, obesity also increases the risk of future cardiovascular events. Nevertheless, some research has pointed out that certain groups of obese patients may fare better, the so-called “obesity paradox” [3], and likewise have questioned the importance of obesity without metabolic abnormalities (“metabolically healthy obesity”) on the development of future adverse CV events [1].

The metabolic syndrome has been used to identify individuals who have a cluster of risk factors, which together pose a higher prevalence of CAD and increased risk of subsequent clinical cardiovascular disease (CVD). The increased CVD risk appears to be related to the risk factor clustering and insulin resistance associated with the metabolic syndrome rather than simply to obesity [4–7]. Moreover, individuals with MetS have a higher risk of CAD when coronary artery calcium (CAC) is increased [8].

The independent association between obesity and cardiovascular disease, particularly coronary artery disease remains controversial. A recent meta-analysis found that compared with metabolically healthy normal-weight individuals, obese individuals are at increased risk for adverse long-term CVD events even in the absence of metabolic abnormalities, suggesting that there is no healthy pattern of increased weight [9]. However, previous studies also demonstrated that metabolic health was the main determinant risk of acute myocardial infarction (MI) [1] or CV outcomes, not obesity itself [10].

The presence of coronary artery atherosclerosis is an established marker of future CV risk in symptomatic individuals [11]. However, the association of those findings according to obesity and presence of MetS and their association with clinical outcomes has not been rigorously studied. Thus, in the present study we sought to investigate the interplay of BMI, MetS and CAD detected by coronary computed tomography and their association with future CV events.

## Methods

### Study population

The methods of the Partners Healthcare CT Registry have been previously described [11, 12]. In brief, we included all consecutive subjects older than 18 years of age who underwent a contrast enhanced cardiac computed tomography angiography (CTA) for the evaluation of the coronary arteries at the Massachusetts General Hospital or the Brigham and Women’s Hospital from 2004–2011. All CTA scans were performed with 64 detector or newer generation scanners. We excluded asymptomatic patients referred for screening purposes or research protocols and patients with prior known CAD [defined as prior percutaneous coronary intervention (PCI), coronary artery bypass graft surgery (CABG), or MI]. The Partners’ Healthcare Institution Review Board approved the study.

### Coronary CTA exam acquisition and interpretation

Coronary CTA were conducted according to published guidelines [13, 14] and institutional protocols at the time of scan acquisition. CTA results were ordinally categorized as having no (0%), non-obstructive (<50%), or obstructive (≥50%) coronary artery disease (CAD). Vessels less than 2 mm luminal diameter were not evaluated. We used the 18 segment coronary model proposed by the American Heart Association [14] to categorize CAD presence, extent, and severity for each segment. More detailed analysis of the extent and severity of CAD were performed using previously validated scores:

- Segment involvement score (SIS): the sum of the number of segments with any plaque, which ranges from 0 to 17 [15].
- Segment severity score (SSS): each segment receives a value according the amount of disease present in that vessel (0: no CAD, 1: non-obstructive CAD, 2: 50–70% stenosis, 3: >70% stenosis). The final score is the sum of each individual score, and ranges from zero to 51 [16].

#### Baseline risk factors

We reviewed all clinical data prior to the coronary CTA to verify the presence or absence of risk factors [17, 18]. We defined hypertension using the most recent definition for components of metabolic syndrome, as a systolic blood pressure ≥130 mmHg, diastolic blood pressure ≥85 mmHg, or diagnosis/treatment of hypertension. We defined obesity as body mass index ≥30 kg/m^2^. We defined hypertriglyceridemia as triglycerides ≥150 mg/dL. We defined low high density lipoprotein cholesterol (HDL) as <40 mg/dL (male) or HDL <50 mg/dL (women). We defined type 2 diabetes mellitus (T2DM) as a hemoglobin A1C (HbA1c) ≥6.5% (45 mmol/mol) [19], two fasting glucose levels ≥126 mg/dL, or diagnosis/treatment of T2DM. We defined dysglycemia as HbA1C ≥ 5.7% (39 mmol/mol) and <6.5% (45 mmol/mol) or fasting glucose level 100–125 mg/dL without known T2DM [19]. We categorized smoking as never, former, or current (tobacco within the last month). We defined family history of premature CAD as any first-degree family member with clinical CAD prior to age 60.

For each patient, we determined the total number of cardiometabolic (CM) risk factors present, since clustering of these common risk factors have been recognized as the key contributors to the pathogenesis of both T2DM and cardiovascular disease [20, 21]. We used the following CM risk factors: (1) obesity; (2) low HDL; (3) hypertriglyceridemia; (4) hypertension; and, (5) dysglycemia. All patients were verified as having T2DM or not according to the criteria listed above. BMI was used as a measure of central adiposity (instead of waist circumference), although these two measures have consistently been shown to be highly correlated [22] and have similar predictive value for future onset of diabetes [23] or clinical CV disease [24]. This method is consistent with the recommendations of the American Association of Clinical Endocrinologists [25] and the World Health Organization [26], both of which include BMI as a means of defining CM risk.

### Cardiovascular outcomes

Two cardiologists blinded to CTA results reviewed all patient medical records for adjudication of cardiovascular events. We mailed a standardized questionnaire to each patient in order to ensure that events outside of our healthcare network were captured. For patients who did not return the questionnaire after repeated mailings, we conducted scripted phone interviews. In addition, patients had the option of completing a web-based version of the questionnaire via the REDCap (Research Electronic Data Capture) system [27], which is secure, encrypted, and HIPAA compliant. Two cardiologists blinded to CTA results verified each self-reported event via outside medical record review. Discrepancies were adjudicated by consensus.

We defined major adverse cardiovascular events (MACE) as a composite of non-fatal myocardial infarction, late coronary revascularization (>90 days), recurrent unstable angina requiring emergency or inpatient hospital evaluation, and cardiovascular death. We additionally evaluated the outcome of cardiovascular death and non-fatal MI to avoid inherent bias of softer outcomes (e.g. angina, coronary revascularization). Diagnosis of MI was confirmed by two of three: chest pain or equivalent symptom complex; positive cardiac biomarkers; ECG changes typical of MI [28]. Time to the first coronary revascularization procedure (PCI or CABG) was evaluated. Early revascularizations (≤90 days post coronary CTA) were removed from survival analysis to minimize verification bias [29–31]. That is, patients with ≥50% stenosis by coronary CTA more frequently undergo invasive angiography and revascularization early after the coronary CTA, whereas symptom driven late coronary revascularizations are less likely to be associated with the coronary CTA and more associated with CAD progression and prognosis. Unstable angina without revascularization (USA) was defined as chest pain or chest pain equivalent with dynamic electrocardiogram (ECG) changes such as ST depression or T wave inversion but without abnormal cardiac biomarkers and characterized by: (1) rest symptoms; (2) new onset angina (less than 2 months duration); or, (3) increasing duration or severity of previously stable anginal symptoms [32].

We confirmed all deaths using the Social Security Death Index. For all patients who died, the cause of death was obtained using the National Death Index as well as the Massachusetts Department of Vital Statistics, when applicable. In addition, other relevant clinical records (e.g. death notes, autopsy findings, hospice notes) related to death were reviewed. Using all available data, two cardiologists blinded to the CTA results adjudicated cause of death, with cardiovascular death defined as a primary cause of acute MI, atherosclerotic coronary vascular disease, congestive heart failure, valvular heart disease, arrhythmic heart disease, stroke, or other structural or primary cardiac cause of death.

### Statistical analysis

Continuous variables are reported as mean and standard deviation. Categorical variables are reported as counts and proportions. Continuous variables were compared between groups using analysis of variance techniques. Median segment scores were compared between groups using the Kruskal–Wallis test. Categorical variables were compared using Chi squared test or Fisher’s exact test, where appropriate. The Kaplan–Meier method and log rank test were used to assess event-free survival for all outcomes of interest. Univariable and multivariable Cox proportional hazards models were constructed to compare risk between strata. The assumption of proportional hazards was tested by the scaled and unscaled Schoenfeld residuals and resulted in non-significant findings in in all analyses. In order to maximize statistical power of the BMI analysis, we further evaluated the full range of BMI as a non-linear J-shaped curve using a second degree multivariable fractional polynomial method [33]. Fractional polynomials allow for nonlinear modeling through selection of transformation powers of independent variables that allow for the most parsimonious best model fit. We used the FRACPOLY command in Stata version 12.1 (Statacorp, College Station, TX, USA). We evaluated for interactions between the transformed BMI and MetS in the fully adjusted Cox regression model. Hochberg or Holm’s corrections for multiple comparisons were applied wherever applicable. All statistics were performed using Stata.

## Results

### Study population

3884 patients underwent CTA during the study period, with 773 having no BMI data, 10 had incomplete clinical outcomes data, 117 had prior CAD, 20 had did not have fully evaluable CTA, and 1846 were lacking risk factor data to define smoking status or MetS. Therefore, the study population consisted of 1118 individuals who met all inclusion criteria and had complete baseline and follow-up data.

### Baseline characteristics

As noted on Table 1, the study population was predominantly male (58%) with a mean BMI of 30 ± 7 kg/m^2^ and increasing prevalence of baseline cardiovascular risk factors with increasing BMI. 133 (22%) subjects without MetS met criteria for T2DM and 318 (63%) subjects with MetS met criteria for T2DM. BMI was categorized as <20 kg/m^2^ (n = 22, 2%), 20–24.9 kg/m^2^ (n = 206, 18%), 25–29.9 kg/m^2^ (n = 423, 38%), 30–39.9 (n = 372, 33%), and >40 kg/m^2^ (n = 95, 8%). Age and smoking did not correlate directly with BMI, although those with BMI > 40 kg/m^2^ were more likely to be current smokers (17 versus 11% overall) and those with BMI < 20 kg/m^2^ were less likely (5 versus 11% overall).

**Table 1:** Baseline characteristics

BMI (kg/m^2^)	<20	20–24.9	25–29.9	30–39.9	>40	All	*p* value
n	22	206	423	372	95	1118	
Age	56 ± 15	57 ± 14	57 ± 13	56 ± 13	54 ± 12	57 ± 13	0.23
Male	5 (23%)	97 (47%)	279 (66%)	216 (58%)	49 (52%)	648 (58%)	<0.0001
Hypertension	7 (32%)	87 (42%)	212 (50%)	249 (67%)	76 (80%)	626 (56%)	<0.0001
Dysglycemia	15 (68%)	119 (58%)	271 (64%)	283 (76%)	79 (83%)	760 (68%)	<0.0001
Diabetes	8 (36%)	55 (27%)	140 (33%)	192 (52%)	56 (59%)	451 (40%)	<0.001
Elevated TG	3 (14%)	33 (16%)	110 (26%)	145 (39%)	42 (44%)	335 (30%)	<0.0001
Low HDL	9 (41%)	37 (18%)	127 (30%)	182 (49%)	58 (61%)	414 (37%)	<0.0001
Metabolic syndrome	4 (18%)	28 (14%)	98 (23%)	286 (77%)	86 (91%)	372 (33%)	<0.001
Current smoker	1 (5%)	25 (12%)	42 (10%)	37 (10%)	16 (17%)	123 (11%)	0.19
Family history	4 (20%)	91 (44%)	195 (46%)	156 (42%)	35 (37%)	481 (43%)	<0.001
CCTA results							<0.0001
No CAD	11 (50%)	103 (50%)	157 (37%)	138 (37%)	28 (29%)	436 (39%)	
<50%	9 (41%)	72 (35%)	157 (37%)	145 (39%)	45 (47%)	425 (38%)	
>50%	2 (9%)	31 (15%)	110 (26%)	89 (24%)	22 (23%)	257 (23%)	

*BMI* body mass index, *CAD* coronary artery disease, *CCTA* coronary CT angiography, *HDL* high-density lipoprotein cholesterol, *SIS* segment involvement score, *TG* triglycerides

During a median 3.2 (IQR 1.9–4.5) years follow-up, there were 46 (4.1%) all-cause deaths, 21 (1.9%) cardiovascular deaths, 13 (1.2%) non-fatal MI, 13 (1.2%) unstable angina without revascularization, and 34 (3.1%) late coronary revascularizations.

### Prevalence, extent, and severity of CAD more strongly associated with metabolic syndrome rather than increasing BMI

**Fig. 1:**
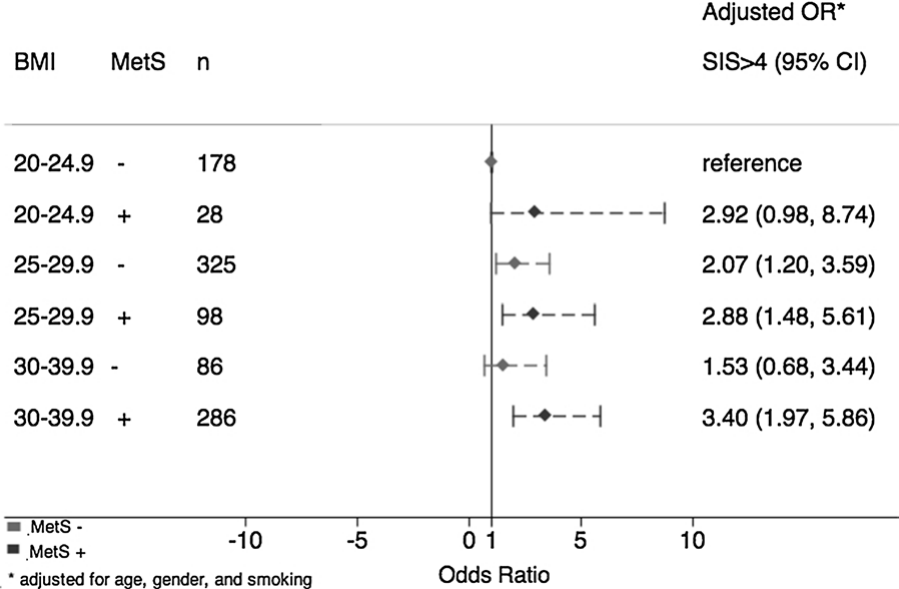
Odds of extensive CAD (Segment Involvement Score, SIS > 4), according to MetS and BMI. *CAD* coronary artery disease, *MetS* metabolic syndrome, *BMI* body mass index, kg/m^2^

By univariable analysis, BMI was associated with presence, extent, and severity of CAD. The univariable odds ratios for each 1 kg/m^2^ increase in BMI as a continuous variable (excluding <20 kg/m^2^) for any CAD, obstructive CAD, extensive CAD, SIS, and SSS were 1.03 (1.01–1.05, p = 0.012), 1.01 (0.99–1.03, p = 0.3), 1.02 (1.0–1.04, p = 0.058), 1.03 (1.01–1.05, p = 0.012), 1.03 (1.01–1.05, p = 0.012), respectively. However, in multivariable analysis after adjustment for age, gender, smoking and each individual metabolic syndrome component, these values were not significant at 1.03 (1.0–1.05, p = 0.06), 1.0 (0.98–1.03, p = 0.8), 1.01 (0.99–1.04, p = 0.2), 1.03 (0.99–1.05, p = 0.06), 1.03 (0.99–1.05, p = 0.06), respectively, suggesting that BMI served as a marker for the components of MetS, which was more significantly associated with CAD than BMI. Each incremental increase in BMI was associated with increasing likelihood of MetS (OR 1.28 per 1 kg/m^2^, p < 0.001). Within each BMI category, when compared to those who were metabolically healthy (−MetS), those who were metabolically unhealthy (+MetS) had greater extent of CAD (Fig. 1).

### Prognosis more strongly associated with metabolic syndrome rather than increasing BMI

By univariable linear analysis, BMI (excluding BMI < 20 kg/m^2^) was not associated with CV MACE, HR = 1.26 (0.99–1.06, p = 0.13) per each 1 kg/m^2^ increase in BMI. Obesity as a binary variable (BMI ≥ 30 kg/m^2^.) was not associated with CV MACE, HR = 1.28 (0.79–2.05, p = 0.31). MetS as a binary variable was associated with CV MACE, HR = 2.09 (1.28–3.40, p = 0.003).

**Fig. 2:**
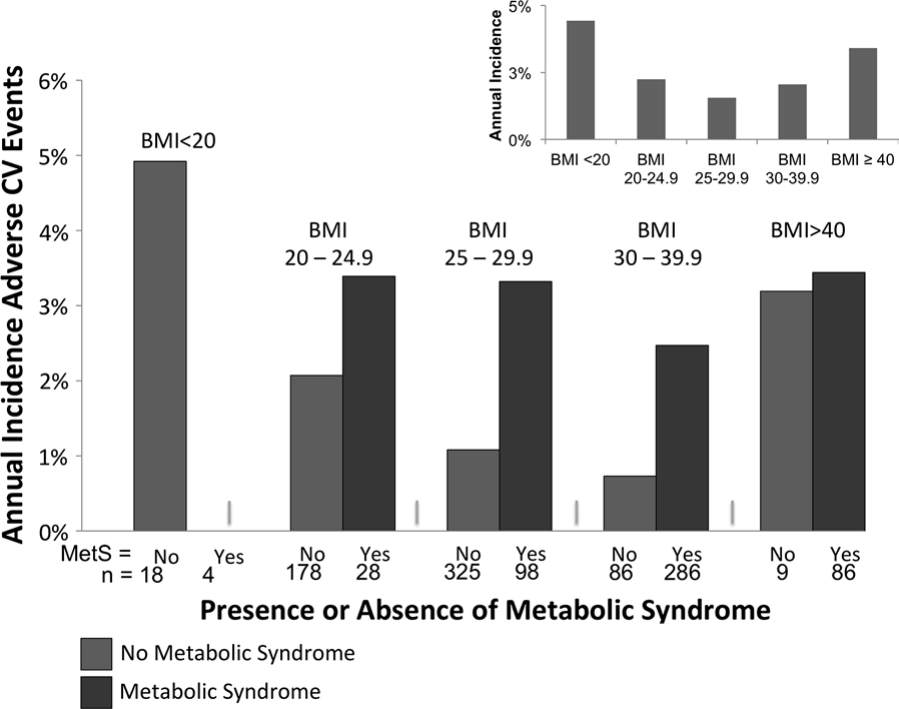
Absolute annualized adverse CV events demonstrated a J-shaped curve according to BMI (*inset*). The presence of MetS (*dark bar*) predicted worse outcomes within the BMI range of 20–39.9 kg/m^2^. *CV* cardiovascular, *MetS* metabolic syndrome, *BMI* body mass index, kg/m^2^

Within each BMI category, prognosis was poorer for those who were metabolically unhealthy (+MetS) versus healthy as indicated by increased annualized incidence of adverse CV events (Fig. 2). Univariable and multivariable (adjusted for age, gender and smoking) HR for each BMI category are presented on Table 2 for the endpoint of CV MACE (top panel) and CV death/MI (Table 2, lower panel), although this endpoint included just 31 events and thus was underpowered for stratification by BMI categories.

**Table 2:** Univariable and multivariable hazard ratios for cardiovascular major adverse events (top panel Cardiovascular MACE) and the combined events of cardiovascular death or MI (lower rows CV Death or MI)

Predictor	Univariable			Multivariable		
Variable	HR	95% CI	p value	HR	95% CI	p value
Cardiovascular MACE (CV death, non-fatal MI, late coronary revascularization, and unstable angina)						
BMI, kg/m^2^	1.03	0.99–1.06	0.13	1.04	1.01–1.08	0.02
Obesity	1.28	0.79–2.05	0.31	1.45	0.9–2.34	0.13
MetS	2.09	1.28–3.4	0.003	1.88	1.15–3.08	0.01
BMI 20-24.9, MetS−	1	Reference		1	Reference	
BMI 20-24.9, MetS+	1.67	0.47–5.93	0.43	1.52	0.43–5.39	0.52
BMI 25-29.9, MetS−	0.5	0.22–1.14	0.1	0.51	0.22–1.17	0.11
BMI 25-29.9, MetS+	1.56	0.65–3.7	0.32	1.12	0.47–2.69	0.8
BMI 30-39.9, MetS−	0.31	0.07–1.41	0.13	0.38	0.08–1.74	0.21
BMI 30-39.9, MetS+	1.13	0.56–2.3	0.73	1.15	0.56–2.33	0.71
CV death or MI						
BMI, kg/m^2^	1.01	0.96–1.06	0.7	1.03	0.98–1.09	0.25
Obesity	1.44	0.71–2.91	0.31	1.81	0.88–3.71	0.11
MetS	2.61	1.23–5.55	0.01	2.19	1.02–4.67	0.04

Multivariable models were further adjusted for age, gender, and current smoking. *BMI* body mass index, *CV* cardiovascular, *HR* hazard ratio, *MetS* metabolic syndrome, *MI* myocardial infarction

**Fig. 3:**
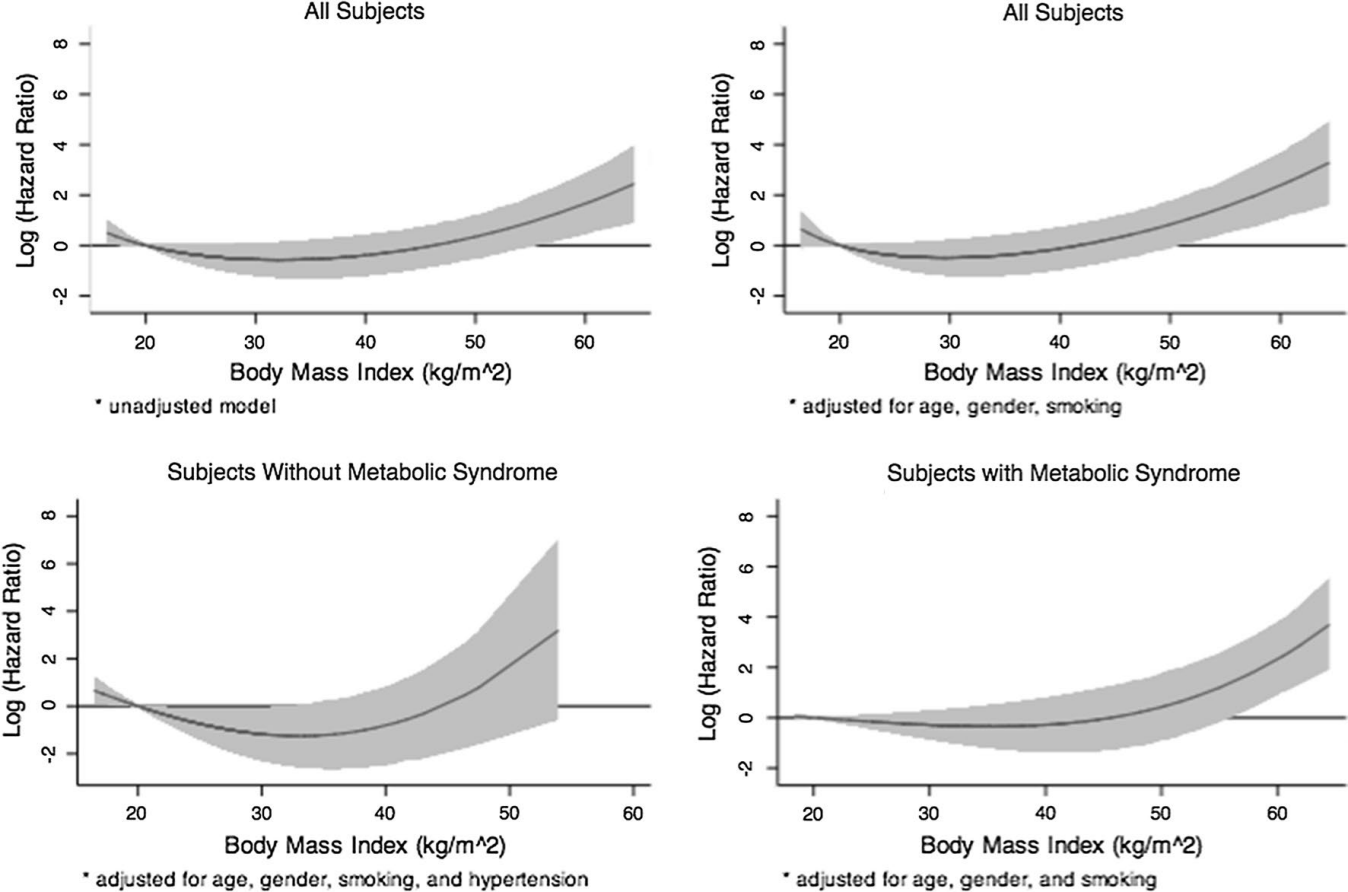
Log hazard ratio according to body mass index (kg/m^2^) for cardiovascular adverse events (cardiovascular death, nonfatal MI, unstable angina requiring hospitalization, or coronary revascularization >90 days post-CTA). HR are unadjusted (*top left*), adjusted for all patients (*top right*) and adjusted and stratified by absence (*bottom left panel*) or presence (*bottom right panel*) of metabolic syndrome (MetS). Reference BMI was set at 20 kg/m^2^. An “obesity paradox,” where patients with relatively increased BMI from 20–40 kg/m^2^ had lower hazard of adverse CV events was observed particularly in the subjects without MetS, which may indicate residual confounding

**Fig. 4:**
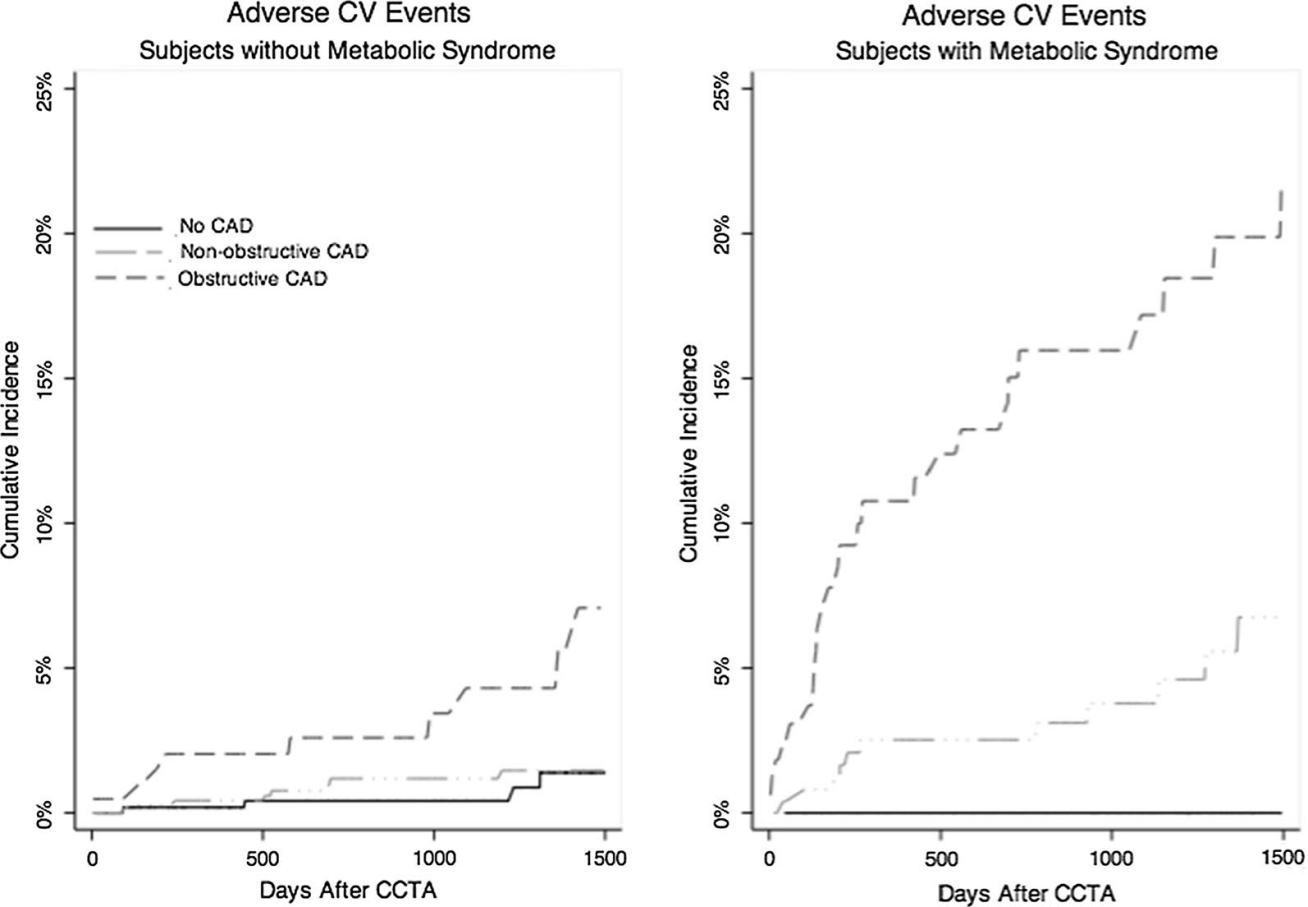
Cumulative incidence of adverse cardiovascular (CV) events (CV death, nonfatal MI, unstable angina requiring hospitalization, or coronary revascularization >90 days post-CTA), stratified by no MetS (*left panel*) and MetS (*right panel*) and normal CTA versus non-obstructive CAD (*short dash line*) versus obstructive CAD (*long dash*). Overall, patients with MetS experienced outcomes over 2× the rate of those without. Those with MetS and obstructive CAD experienced very high adverse events. The *left panel* was adjusted as in Fig. 3 for BMI, age, gender, current smoking, and hypertension. The *right panel* was adjusted as in Fig. 3 for BMI, age, gender, and current hypertension

To evaluate the full range of BMI as a non-linear J-shaped curve, we first evaluated BMI as a predictor of CV MACE in a univariable second degree fractional polynomial model (Fig. 3, top left). The best fit model included BMI^−1^ and BMI^3^, with HR = 182 (2–15675, p = 0.02) and HR = 1.02 (1.001–1.03, p < 0.001), respectively. We then adjusted for age, gender, and current smoking (Fig. 3, top right). The best fit model included BMI^−2^ and BMI^3^, with HR = 1395 (6–313,075, p = 0.009) and HR = 1.02 (1.01–1.03, p < 0.001), respectively. After stratification by MetS, we modeled those without MetS by adjusting first by BMI, age, gender, and smoking and then incrementally evaluating each of the individual components of MetS (low HDL, hypertension, dysglycemia, elevated TG) for significance. The best fit model included BMI^2^, BMI^3^, age, gender, current smoking, and hypertension (Figure 3, bottom left), with HR for BMI = 0.42 (0.21–0.82, p = 0.01) and HR = 1.19 (1.05–1.34, p = 0.008), respectively. Last, we modeled those with MetS and adjusted for age, gender and current smoking (Fig. 3, bottom right). The best fit model included BMI^3^, BMI^3^, age, gender, and current smoking, with HR for BMI = 0.93 (0.85–1.01, p = 0.095) and HR = 1.05 (1.0–1.10, p = 0.05), respectively. The cumulative incidence of CV MACE for those with and without MetS could be further stratified according to CTA finding, as demonstrated in Fig. 4.

In order to evaluate for other relationship of BMI with MetS, we included in the fractional polynomial Cox regression model a test for interaction between BMI and MetS that was not significant in all analyses, indicating no additional influence of BMI upon MetS as a predictor of CV MACE.

## Discussion

Our study has demonstrated that metabolic risk modifies the association of BMI with increasing prevalence, extent, and severity of CAD. Metabolic risk among BMI categories also modified the risk of incident cardiovascular events. These findings suggest that although BMI serves as a marker of CAD risk when considered in isolation, the hazard associated with BMI is mostly mediated by the presence of other metabolic risk factors. Our group has previously demonstrated an incremental increase in CAD burden and future adverse events with incrementally worse metabolic health [7]; the current study extends upon the prior analysis by modeling CV prognosis non-linearly across the full range of BMI. Although most obese individuals have a cluster of CM risk factors, those without this pattern have a significantly lower probability of CAD and future CV events. Therefore, one must carefully evaluate all potential risk factors when considering the presence or absence of an “obesity paradox” since not all obesity phenotypes have a homogenous prevalence of CAD or risk of adverse CHD events.

In prior studies, a variety of authors have suggested evidence of an “obesity paradox,” yet others have discredited this finding as an effect of residual confounding. For example, Lavie et al. [3] have noted that since heart failure is a catabolic state, obese patients have improved survival relative to normal weight, which may simply demonstrate residual confounding due to more advanced disease state. Similar findings of “obesity paradox” have been reported with hypertensive patients, although this may relate to different pathophysiology and co-morbid conditions among the obese versus normal weight with hypertension [3]. Despite such counter-intuitive reports, a large pooled analysis of 19 prospective studies that excluded smokers and those with known cancer or heart disease identified no evidence of obesity paradox [34]. In this study, a J-shaped curve for obesity in association with all-cause death was noted and the healthiest BMI was noted to be 20–24.9 kg/m^2^. Each incremental 5 point increase in BMI was associated with a hazard ratio of 1.31 (95% CI, 1.29–1.33) [34]. Similarly, the Prospective Studies Collaboration identified the healthiest BMI as 22–25 kg/m^2^ for the lowest association with cardiovascular death with a hazard ratio per 5 point increase of 1.32 (95% CI, 1.29–1.36) [35]. An important large study of 13,874 patients undergoing coronary CTA reported greater prevalence, extent and severity of CAD in overweight and obese individuals. BMI was independently associated with a higher risk of non-fatal MI. Although BMI was not associated independently with all-cause death, this study was limited by linear modeling of BMI as a risk factor without consideration of modeling effects of extreme BMI < 20 kg/m^2^ or BMI > 40 kg/m^2^ [36].

An emerging phenotype of “metabolically healthy obesity (MHO)” has been recognized, although the literature to date is not comprehensive. A recent systematic review on the topic of metabolically healthy obesity identified 15 cohort and 5 cross-sectional studies that defined metabolically healthy obesity using either lack of insulin resistance or lack of MetS [37]. This review noted that only two of seven cohort studies that evaluated all-cause death found a significantly increased risk, one of seven evaluating CV deaths, and 3 of 9 evaluating incident CVD. However, 5 of 9 evaluating incident CVD demonstrated a consistent trend for MHO to have an increased clinical risk relative to metabolically healthy normal weight, while only 1 of 9 studies showed no association whatsoever. Thus, although the available studies to date are not numerous, the majority of cohort studies published have not consistently proven that metabolically healthy obesity is not associated with increased adverse clinical outcomes when compared to metabolically healthy normal weight individuals (in contrast to metabolically unhealthy obesity which does convey worse prognosis). However, 5 cross-sectional studies suggested a slight increase in subclinical atherosclerosis among those with MHO, which suggests that with a longer follow-up and larger sample size a small increased risk might be identified even for metabolically healthy obesity. A study looking at progression of plaque with coronary CTA reported that MetS was an independent predictor of progression of coronary artery stenosis or development of vulnerable plaque after accounting for traditional CM risk factors including BMI, HR = 1.47, (95% CI 1.01–2.15, p = 0.045) [38].

Scientists have debated the relative merits of regional adiposity (e.g., abdominal or central obesity) versus absolute weight metrics, such as body mass index. Although most widely applied criteria for MetS diagnosis place greater emphasis on central adiposity, this measure is rarely measured clinically and thus the WHO and others [25, 26], often substitute BMI as a reasonable, albeit imperfect, surrogate. Nevertheless, several studies have demonstrated a relevance of central adiposity. For example, a large screening cross-sectional study of 21,335 middle-aged Korean men identified that abdominal adiposity was associated with CAC, although ultrasound evidence of non-alcoholic fatty liver disease when compared to abdominal obesity, demonstrated an even stronger association for CAC [39]. On the other hand, in a smaller Asian screening cross-sectional study of 3157 subjects who underwent CT, visceral adiposity was associated with CAD in univariable analysis but not by multivariable analysis after adjustment for age, gender, dyslipidemia, diabetes mellitus, and the ratio of visceral to subcutaneous fat [40]. Yet another interesting study stratified 2078 normal weight subjects (18.5 ≤ BMI < 25 kg/m^2^) who underwent CTA according to percentage of body fat. Individuals with the highest tertile of body fat, even at normal weight (“normal weight obesity”) exhibited increased prevalence of CTA non-calcified coronary plaques (21.6 vs. 14.5%, p = 0.039), more abnormal aortic pulse wave velocity, and increasingly abnormal cardiometabolic risk factors [41]. Thus, although not routinely measured in most clinics, several reports have demonstrated a value of evaluating central adiposity.

Currently over half of American adults are overweight or obese. Furthermore, in contrast to previous decades there is a shift toward a greater proportion of morbid versus milder obesity [3]. Some encouraging recent data suggest a plateau in the prevalence of American obesity, yet the prevalence worldwide has continued to increase in almost every corner of the globe, with certain regions particularly afflicted, such as parts of India [42]. For example, the worldwide prevalence of obesity has doubled from 1980 to 2008, such that over 1.4 billion adults are now overweight and a 0.5 billion are obese. Even more concerning is the crisis of childhood obesity with 43 million preschoolers [43] worldwide overweight, who may begin to have adverse cardiovascular structural and metabolic effects even in childhood and early teen years [44]. Obese individuals are at increased risk of diabetes, heart disease, stroke, sleep apnea, and other chronic conditions and consume increased healthcare resources relative to those of normal weight.

In spite of the debate about an “obesity paradox,” this often represents a comparison to unhealthy thinner patients in an end-stage disease state (as in CHF), residual confounding (such as when smoking is not adequately accounted for), or overly adjusting for co-linear variables associated with obesity (such as dyslipidemia, dysglycemia, or hypertension). Because of the important health effects of obesity as demonstrated by our study and others, for conditions including CAD, obstructive sleep apnea, heart failure, stroke, or death, successful weight loss interventions are of great importance. Although guidelines endorse exercise and healthy as first line recommendations, long term success through lifestyle interventions remain elusive for many patients. Recently, the 3 year follow-up results of the STAMPEDE trial, which compared bariatric surgery to non-surgical intervention for obese patients with uncontrolled type 2 diabetes mellitus and noted persisting improvements in metabolic health and weight loss after for surgery [45]. Thus, identifying patients with MetS, which may be treatable and in some cases reversible through lifestyle or surgical intervention, could benefit lifetime cardiovascular risk.

Contemporary interest to improve risk stratification of patients with either MetS or diabetes mellitus have led to limited studies of screening populations. For example, although major guidelines define diabetes mellitus as high-risk for CAD events, one study screened 98 asymptomatic patients with diabetes using CAC, CTA, and carotid ultrasound and identified 55 (56%) who had no detectable CAD and could potentially be re-classified from high to low CV risk. Sixteen subjects (including three with CAC = 0) were found to have obstructive CAD by CTA, but no clinical outcomes were reported for this cross-sectional study [46]. Similarly, Ryu et al. screened 755 patients with MetS by CTA and identified an increasing extent and burden of CAD according to the number of MetS risk factors, among which abdominal obesity and hypertension had the strongest effect [47]. A small study of 39 Japanese men also noted that those with versus without MetS had higher prevalence of CAD (31.3 versus 4.3%, p = 0.033) and lower serum adiponectin levels (4.5 ± 0.6 versus 6.4 ± 0.6 μg/mL, p = 0.014) [48]. Another study that screened 1000 asymptomatic Korean patients with diabetes found that 78% had no detectable CAD by CTA. Although 22% had some plaque that may modify preventive clinical decision making, the incidence of downstream adverse clinical events was low over 17 months follow-up and consisted entirely of 1 unstable angina and 14 coronary revascularizations [49]. The FACTOR-64 study, which randomized asymptomatic diabetics to screening by CTA or standard therapy also failed to show any significant difference in downstream events, owing to the unexpectedly low number of events and the use of preventive therapies in both arms (>70% on statins) [50]. Thus, although screening can further characterize the otherwise homogenous risk categorization of patients with diabetes from simply “high risk,” the impact of such testing upon the otherwise low clinical event incidence among asymptomatic patients remains uncertain.

Notwithstanding these results, our study must be interpreted in the context of its inherent limitations. First, a retrospective design in a tertiary referral center may result in selection bias. Similarly, the patients were clinically referred for a coronary CTA, which may increase the prevalence of CAD, and clinical risk compared to the general population. Also, we did not have available measures of central adiposity, such as waist circumference. In spite of these limitations, we demonstrate that although obesity is associated with CAD prevalence, extent, severity and prognosis much of the risk can be explained by cardiometabolic health within obesity subcategories.

## Conclusion

Compared to individuals with normal BMI (20–24.9 kg/m^2^), there was an increased prevalence, extent, and severity of CAD for those with BMI > 25 kg/m^2^. BMI by univariable analysis demonstrated a J-shaped curve with prognosis, though this association was significantly modified after adjusting for metabolic risk factors. BMI was a marker for MetS, which was more significantly associated with prognosis.
